# Prospective evaluation of cardiac ultrasound performance by general internal medicine physicians during a 6-month faculty development curriculum

**DOI:** 10.1186/s13089-018-0090-7

**Published:** 2018-04-24

**Authors:** Christopher J. Smith, Abdulrahman Morad, Christopher Balwanz, Elizabeth Lyden, Tabatha Matthias

**Affiliations:** 1 Section of Hospital Medicine, Division of General Internal Medicine, Department of Internal Medicine, University of Nebraska Medical Center, 986430 Nebraska Medical Center, Omaha, NE USA; 2 Division of Cardiology, Department of Internal Medicine, University of Nebraska Medical Center, Omaha, NE USA; 3 Department of Biostatistics, University of Nebraska Medical Center, Omaha, NE USA

**Keywords:** Point-of-care ultrasound, Faculty development, Internal Medicine

## Abstract

**Background:**

Point-of-care (POCUS) education is rapidly expanding within medical schools and internal medicine residency programs, but lack of trained faculty is a major barrier. While POCUS training can improve short-term outcomes, knowledge and skills rapidly decay without deliberate practice and feedback. The purpose of this study was to evaluate the performance of focused cardiac ultrasound (FCU) by volunteer general internal medicine (GIM) faculty participating in a longitudinal POCUS curriculum.

**Methods:**

Participants: Nine GIM clinician-educators participated in a 6-month POCUS curriculum. Faculty performance was compared to three cardiology fellows. Three diagnostic cardiac sonographers (DCS) were also evaluated and served as the gold standard. Evaluation: the primary outcome was a FCU efficiency score, calculated by dividing image quality score by exam duration. FCU exams were conducted on three standardized patients after completion of an introductory workshop, at 3 months, and at 6 months. Two blinded cardiologists scored the exams. Analysis: mean efficiency scores were compared using a linear mixed effects model, followed by pairwise comparisons using Tukey’s test.

**Results:**

GIM faculty’s FCU efficiency scores were maintained over the 6-month period (2.2, SE 1.0 vs. 3.8, SE 1.0, *p* = 0.076). Their scores at each session were similar to cardiology fellows (*p* > 0.69), but inferior to DCSs (*p* < 0.0001).

**Conclusion:**

GIM faculty participating in a POCUS curriculum maintained their FCU performance over 6 months with efficiency scores comparable to experienced cardiology fellows.

**Electronic supplementary material:**

The online version of this article (10.1186/s13089-018-0090-7) contains supplementary material, which is available to authorized users.

## Background

Point-of-care ultrasound (POCUS) is clinician-performed ultrasonography used to guide real-time diagnostic and management decisions. POCUS training is rapidly expanding in undergraduate and graduate medical education. Twenty-eight percent of medical schools offer POCUS curricula [1], including several well-established, vertically-integrated programs [2–4]. A survey of Association of Program Directors in Internal Medicine members found that 25% of respondents reported formal POCUS curricula, with another 25% planning to implement programs within a year [5]. Educational leaders in these studies identified faculty development as a major barrier to broader adoption of POCUS training [1, 5].

Despite the increasing need for POCUS-trained faculty in medical education, limited research has investigated training programs for general internal medicine (GIM) faculty. One 10-week faculty program resulted in improved self-reported confidence and exam scores, but image acquisition skills were not assessed [6].

POCUS training can improve short-term outcomes, but knowledge and skills decay without ongoing support. A study of internal medicine physicians who completed a 3-year POCUS curriculum during residency demonstrated poor retention of cardiac ultrasound skills after more than 1 year of nonuse [7]. Surgical residents and medical students who participated in a 2-h cardiac ultrasound course had significant decay in their knowledge and skills within 1 month [8]. Studies of internal medicine residents found knowledge deteriorated in the months following workshop-based training [9], but that longitudinal support prevented knowledge decay [10].

This study investigated the impact of a longitudinal POCUS curriculum on GIM faculty’s performance of focused cardiac ultrasound (FCU). We hypothesized that at the end of the study period, GIM faculty performance of FCU would be resilient to skills decay and comparable to cardiology fellows’ performance.

## Methods

### Participants and setting

**Fig. 1:**
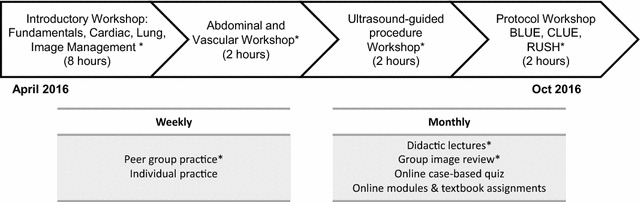
Curricular elements of 6-month point-of-care ultrasound curriculum for internal medicine faculty. *Curricular elements that qualified for CME credits. *BLUE* bedside lung ultrasound in emergency, *CLUE* cardiopulmonary limited ultrasound examination, *RUSH* rapid ultrasound for shock and hypotension

Nine volunteer GIM clinician-educators at an academic health center participated in a 6-month POCUS curriculum with a goal of developing core faculty for a residency program. Faculty did not receive protected time from other duties. Figure 1 illustrates the structure and content of the faculty development curriculum, including elements that were eligible for continuing medical education (CME) credit. The introductory workshop was required, but all other curricular elements were voluntary. Subsequent lectures and workshops were scheduled to accommodate as many participants as possible. Before these sessions, participants were asked to review relevant online modules from free open-access medical education resources. Modules were typically 10–15 min in duration. In-person didactics were recorded and posted to an online learning management system for asynchronous review. Peer-group practice was arranged by group members.

GIM faculty performance was compared to three senior cardiology fellows who had completed level 1 echocardiography training [11], including performance of 75 comprehensive echocardiographic studies. Three registered diagnostic cardiac sonographers (DCS) served as the gold standard.

### Evaluation

We scored FCU exams using a validated image acquisition assessment tool [12]. As in the validation study, our primary outcome was an FCU efficiency score, calculated by dividing an image quality score by exam duration in minutes. The original 68-point image quality score was modified to 66-points, as two scoring items were not taught (inferior vena cava M-mode and respiratory variation measurement). The scoring instrument was comprised of two sections. The first section included scoring of anatomic structures and image optimization (appropriate depth, gain, and centering) in the following views: parasternal long axis; parasternal short-axis aortic valve, mitral valve, mid-papillary, and apex; apical four chamber; subcostal long axis; and subcostal inferior vena cava (IVC). The second section scored the overall diagnostic image quality of the exam for common clinical questions, such as left ventricular systolic function. Assessments took place after completion of an 8-h introductory workshop (baseline), at 3 months, and at 6 months. FCU exams were performed on the same three standardized patients (SPs) at each session. The research team chose SPs that represented a cross-section of typical IM patients (two women, age 48–79 years, body mass index 23–39 kg/m^2^). One SP had a hiatal hernia, which was not known prior to the research sessions. Using cart-sized point-of-care ultrasound machines (Sparq; Philips Healthcare, Andover, MA), participants captured video loops in the required views, outlined above. Before each session, written instructions were provided to participants, facilitators, and SPs (Additional file 1: Appendix S1). The machines used for the assessment were the same as those available to GIM faculty during their curriculum. Before each assessment session, the cardiology fellows and DCS received an in-person tutorial on machine set-up and knobology. Two board-certified cardiologists scored the FCU exams. They were blinded to participant group, SP, and exam session. Prior to scoring research images, they scored three pilot exams together to ensure similar application of the scoring instrument. They then independently scored two pilot exams, resulting in nearly identical scores (53 vs. 52 and 41.5 vs. 42.5 points).

### Analysis

Mean FCU efficiency scores, image quality total score, image quality sub-group scores, and exam duration were compared using a linear mixed effects model with random effects for SP and participant and fixed effects for session and group (GIM faculty, cardiology fellows, and DCSs). If the interaction of group and session were statistically significant, Tukey’s test was performed to make pairwise comparisons of the mean scores between the three groups for each session. If the interaction of group and session was not significant, pairwise comparisons were performed between the groups (combining sessions) and between the sessions (combining groups). Model adjusted means and standard errors (SE) were used for descriptive statistics. Spearman correlation coefficients were used to assess the association of total CME hours with efficiency score. *p* < 0.05 was considered statistically significant. Statistics were calculated using SAS Version 9.4 software (SAS Inc., Cary, NC). The University of Nebraska Medical Center Institutional Review Board approved the study (163-16-EX).

## Results

**Fig. 2:**
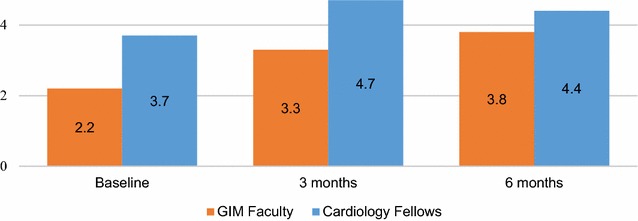
Focused cardiac ultrasound efficiency scores for GIM faculty and cardiology fellows. Baseline line assessment occurred after GIM faculty completed an introductory point-of-care ultrasound workshop. There were no significant differences between the two groups at any of the three assessments

Four of nine GIM faculty were female (44%) and eight (89%) had no prior POCUS training. Eight participants (89%) completed all three sessions. Faculty earned an average of 30.6 CME hours (SD 8.6 h) over the 6 months. The average attendance at voluntary curricular components (workshops, didactics, and image review sessions) over the 6 months was 68% (range 38–88%). FCU efficiency score data are displayed in Table 1. GIM faculty efficiency scores were maintained over the 6-month study period (baseline 2.2, SE 1.0 vs. 6 months 3.8, SE 1.0, *p* = 0.076). There was no difference between mean GIM faculty and cardiology fellow efficiency scores during any of the three assessment sessions (Fig. 2). DCSs performed significantly better than GIM faculty and cardiology fellows during all three assessments (*p* < 0.0001).

**Table 1:** Focused cardiac ultrasound efficiency scores for participant groups throughout the 6-month study period

Baseline			3 months			6 months		
GIM	CF	*p*	GIM	CF	*p*	GIM	CF	*p*
2.2 (1.0)	3.7 (1.2)	0.69	3.3 (1.0)	4.7 (1.2)	0.77	3.8 (1.0)	4.4 (1.2)	1.00

GIM	DCS	*p*	GIM	DCS	*p*	GIM	DCS	*p*
2.2 (1.0)	11.7 (1.2)	< 0.001	3.3 (1.01)	16.0 (1.2)	< 0.001	3.8 (1.0)	14.3 (1.2)	< 0.001

CF	DCS	*p*	CF	DCS	*p*	CF	DCS	*p*
3.7 (1.2)	11.7 (1.2)	< 0.001	4.7 (1.16)	16.0 (1.2)	< 0.001	4.4 (1.2)	14.32 (1.2)	< 0.001

Data reported as model-adjusted means (SE). Comparisons reported using adjusted *p* value

*GIM* general internal medicine faculty, *CF* cardiology fellows, *DCS* diagnostic cardiac sonographers, *SE* standard error

GIM faculty image quality scores were sustained between the baseline and 6-month evaluations (36.7, SE 5.2 vs. 39.7, SE 5.3, *p* = .34). In the linear mixed effect model analysis, there was no significant interaction between group and session for image quality score (*p* = 0.73), so pairwise comparison was made combining the three sessions. Overall, cardiology fellows had a higher mean image quality score than GIM faculty (50.6, SE 6.0 vs. 40.8, SE 5.0, *p* = 0.037). Table 2 displays the image quality scores for each exam sub-section. GIM faculty and cardiology fellow sub-group scores were comparable for the parasternal long axis and subcostal long axis views, as well as for overall diagnostic quality. Cardiology fellow scores were significantly higher for the parasternal short axis, apical four chamber, and subcostal IVC.

**Table 2:** Focused cardiac ultrasound image quality scores for general internal medicine (GIM) faculty and cardiology fellows

	Maximum score	GIM faculty	Cardiology fellows	*p* value
Parasternal long	12	8.7 (0.7)	9.6 (0.9)	0.50
Parasternal short	18	11.0 (0.9)	14.5 (1.3)	0.04
Apical	10	6.0 (0.8)	8.1 (1.0)	0.04
Subcostal long	8	4.2 (1.3)	5.2 (1.4)	0.46
Subcostal IVC	6	2.5 (1.0)	3.6 (1.0)	0.03
Diagnostic quality	12	8.6 (1.0)	9.5 (1.2)	0.52
Total	66	40.8^a^ (5.0)	50.6 (6.0)	0.04

Data reported as model-adjusted means (SE) combining the three assessment sessions. Comparisons reported using adjusted *p* value

*GIM* general internal medicine faculty, *SE* standard error

^a^Model adjusted mean reported, so total does not equal sum of individual sub-sections

GIM faculty’s exam duration decreased during the study period (baseline 17.5 min, SE 1.5 vs. 6 months 13.0, SE 1.5 min, *p* < .001). Combining the three sessions, GIM faculty and cardiology fellow exam duration were comparable across the three sessions (faculty 15.3 min, SE 1.4 vs. fellows 13.8 min, SE 1.8, *p* = 0.64).

## Discussion

GIM faculty participating in a voluntary, multi-modal curriculum maintained their FCU acquisition skills over a 6-month period. GIM faculty efficiency scores were similar to those previously reported by critical care physicians with American Society of Echocardiography level III certification (although the latter performed FCU on mechanically ventilated patients) [12]. Our findings highlight the importance of longitudinal support when teaching FCU to novice faculty. FCU performance requires integration of a complex set of psychomotor skills, which cannot be mastered without regular practice and feedback. Prior studies of novice cardiology fellows [13] and hospitalists [14] found that FCU skills can be developed in weeks to months with frequent expert coaching. The duration of training required to achieve proficiency likely depends on institutional resources and needs. In the present study, GIM faculty did not have dedicated time and was concomitantly learning non-cardiac POCUS applications, requiring a longer curriculum.

Our primary outcome was a measure of efficiency, which Gaudet et al. [12] have argued is a “hallmark of expertise”. Efficiency has been used as an assessment variable in prior FCU performance studies [15] and is important for several reasons. First, POCUS must be economical for integration into clinicians’ daily workflow. Exam efficiency is also vital for the prompt assessment of time-sensitive clinical conditions, such as shock. Finally, task efficiency may precede improvements in performance quality. This phenomenon has been observed in prior FCU studies [12, 13] and performance research for other fine-motor skills, such as endoscopy [16].

While GIM faculty had efficiency scores similar to cardiology fellows, their total image quality scores were lower. This reason for this is likely multifactorial. GIM faculty may not have engaged in enough deliberate practice over the 6 months to advance their image acquisition skills [17]. Additionally, cardiology fellows’ extensive experience in interpreting diagnostic echocardiography studies may have contributed to their superior scores. It should also be noted that our FCU protocol included views which are more technically challenging for novices [15]. Some of these views, such as apical four chambers, are excluded from commonly-used FCU exam protocols [18]. Although their total image quality scores were lower, GIM faculty performed comparably to cardiology fellows for overall diagnostic quality. This may be the most clinically relevant outcome, as the primary purpose of POCUS is to quickly gain information to guide clinical decision making.

This study adds to the existing literature in several ways. It is one of the first papers to examine the educational outcomes of a longitudinal POCUS curriculum for GIM faculty. Studies of residents and fellows may not be applicable to faculty, given differences in clinical experience, time availability, and willingness to adopt innovations [19]. Additionally, we assessed performance of a complete FCU exam protocol including all recommend views [20] and utilized a validated scoring instrument. Finally, the baseline assessment occurred *after* an intensive introductory workshop, allowing us to evaluate the impact of the longitudinal portion of the curriculum independent of the introductory training.

There are limitations to this study. It was conducted at a single institution with few participants. Repeated scanning of the same SPs may have led to higher scores due to familiarity, although this was likely limited by the 3-month washout periods. Furthermore, only the GIM faculty group showed improvement across all testing periods. We were unable to accurately track the number of exams performed by each participant during the curriculum to look for correlation with performance outcomes. Additionally, our study did not assess image interpretation skills, which typically develop more quickly than image acquisition skills [21].

In conclusion, GIM faculty participating in a longitudinal POCUS curriculum maintained their FCU acquisition skills over a 6-month period. Efficiency scores were comparable to cardiology fellows, but overall image quality was lower. Future studies will need to examine if GIM performance of FCU is sustainable beyond 6 months.

## Additional file

**Additional file 1.** Instructions for study participants, facilitators, and standardized patients.

## Abbreviations

POCUS: point-of-care ultrasound
GIM: general internal medicine
FCU: focused cardiac ultrasound
CME: continuing medical education
DCS: diagnostic cardiac sonographer
SP: standardized patient
SE: standard error
BLUE: bedside lung ultrasound in emergency
CLUE: cardiopulmonary limited ultrasound examination
RUSH: rapic ultrasound for shock and hypotension
