# Supplementary material for: Prospective evaluation of cardiac ultrasound performance by general internal medicine physicians during a 6-month faculty development curriculum
Source: Crit Ultrasound J. 2018 Apr 24;10:9. doi: 10.1186/s13089-018-0090-7 (PMC5915984; doi:10.1186/s13089-018-0090-7)
Supplement: Supplementary file 1 — Additional file 1. Instructions for study participants, facilitators, and standardized patients. [file 13089_2018_90_MOESM1_ESM.docx]

**Focused Cardiac Ultrasound**

**Image Acquisition Assessment**

**Participant Instructions**

This is an assessment of your image acquisition skills in focused cardiac ultrasound. This will take into account both image quality and efficiency. You will be asked to complete a focused cardiac ultrasound on 3 standardized patients (SP) using the Philips SPARQ machine. Please note the following:

- You will have a maximum of 20 minutes per patient to acquire the best images you can in the following views.
  - Parasternal long-axis
  - Parasternal short-axis aortic valve
  - Parasternal short-axis mitral valve
  - Parasternal short-axis papillary muscle
  - Parasternal short-axis apex
  - Apical 4-chamber
  - Sub-costal long-axis 4-chamber
  - Sub-costal IVC
  - Measurement of IVC diameter (still image)
- You may reposition the SP to optimize your images.
- Adjust depth, gain, and position of anatomic structure to optimize the image.
- Save images to the machine when you feel they are optimized by hitting the “Acquire” button. You may save more than one image per view.
- Tell the SP when you have completed the exam, so they can track your time to completion. Remember that quality and efficiency will be used when evaluating your images, so you may use less than the entire 20 minutes. A timer will display the amount of time remaining in the session. If you are still scanning when the 20 minutes has expired, stop your exam and exit the room.
- When you are done with the exam, exit the exam room and wait outside the door. This will notify the facilitator that you are done.
- When all 3 participants have completed scanning their SPs, the facilitator will reset the machines and direct you to the next room.
- If you have problems during the exam, notify your facilitator.

**Focused Cardiac Ultrasound**

**Image Acquisition Assessment**

**Facilitator Instructions**

Participant throughput:

- Review instructions with the group and answer question.
- Each SP will receive a timer. Set these for 20 minutes and instruct them how to start/stop the timer.
- Once the machine and patients are set-up (see below) and participants are ready, announce the group to begin their exams. At this point, the SPs should start their timer.
- Participants should notify the SP when they complete the exam. The SP should stop the timer.
- The facilitator will keep a back-up timer. When a participant exits the room, document the time on the score sheet in the appropriate column.
- Each participant will wait until the others are done or the 20 minute time limit is reached. There will be a 5 minutes break between each of the 3 exams for you to document times and reset the machines for the next participant. Note the timers used by the SPs count *down* from 20 minutes, so the stop time will be the time remaining (i.e. subtract the time remaining from 20 min to calculate the time to complete).
- Participants will move to the next room where they will prepare to scan the next SP.

Machine and Room Set-up:

- Place the machine and a chair/stool on the right-side of the exam table. Ensure there is enough room for the participant. We will not be using the EKG leads.
- Have the SP untie their gown. Place them in a neutral supine position on the exam table with a pillow available. Participants will be responsible for repositioning the SPs to optimize image quality.
- Press “Start/End” button, the choose “New Patient” to create a new study
- For the “Last (Family)” name enter the appropriate ID for the standardized patient: SP1, SP2, or SP3.
- For “Performed by” enter the 3-digit identifier for the subject (see below)
- No other information needs to be entered on the “Patient” menu
- Choose S4-2 Transducer
- Choose the Cardiac IM Exam, then select “Save & Exit.”
- Ensure the SP is ready and positioned rotated to left side with pillow under right shoulder

**Focused Cardiac Ultrasound**

**Image Acquisition Assessment**

**Standardized Patient Instructions**

This is an assessment of physician’s image acquisition skills in focused cardiac ultrasound. You will be serving as a standardized patient (SP) for participants to demonstrate the ultrasound exam skills. Please note the following:

- Please remove your clothing above the waist and put on a patient gown. Participants will need to capture images in several locations on your chest and upper abdomen.
- You will be asked to be positioned on your left side and back for the exam. Please do your best to follow requests by the participants to change your position.
- You will be asked to time how long each participant takes to complete their exam. A timer will be provided. Start the timer when instructed by the facilitator at the beginning of the exam. Stop the timer when the participant tells you they are done. The maximum amount of time per exam is 20 minutes.
- When the participant has completed the exam and the timer is stopped, they will leave the room. The facilitator will document the time remaining on your timer and reset the machine for the next exam.
